# Do two and three year old children use an incremental first-NP-as-agent bias to process active transitive and passive sentences?: A permutation analysis

**DOI:** 10.1371/journal.pone.0186129

**Published:** 2017-10-19

**Authors:** Kirsten Abbot-Smith, Franklin Chang, Caroline Rowland, Heather Ferguson, Julian Pine

**Affiliations:** 1 School of Psychology, University of Kent, Canterbury, United Kingdom; 2 ESRC LuCiD Centre & Department of Psychological Sciences, University of Liverpool, Liverpool, United Kingdom; 3 Max Planck Institute for Psycholinguistics, Nijmegen, Holland; University of Leicester, UNITED KINGDOM

## Abstract

We used eye-tracking to investigate if and when children show an incremental bias to assume that the first noun phrase in a sentence is the agent (first-NP-as-agent bias) while processing the meaning of English active and passive transitive sentences. We also investigated whether children can override this bias to successfully distinguish active from passive sentences, after processing the remainder of the sentence frame. For this second question we used eye-tracking (Study 1) and forced-choice pointing (Study 2). For both studies, we used a paradigm in which participants simultaneously saw two novel actions with reversed agent-patient relations while listening to active and passive sentences. We compared English-speaking 25-month-olds and 41-month-olds in between-subjects sentence structure conditions (Active Transitive Condition vs. Passive Condition). A permutation analysis found that both age groups showed a bias to incrementally map the first noun in a sentence onto an agent role. Regarding the second question, 25-month-olds showed some evidence of distinguishing the two structures in the eye-tracking study. However, the 25-month-olds did not distinguish active from passive sentences in the forced choice pointing task. In contrast, the 41-month-old children did reanalyse their initial first-NP-as-agent bias to the extent that they clearly distinguished between active and passive sentences both in the eye-tracking data and in the pointing task. The results are discussed in relation to the development of syntactic (re)parsing.

## Introduction

In order to learn a language, children have to learn how its syntax maps onto who did what to whom in events. For example, they have to learn that in English active transitives (‘the fireman saved the baby’), the agent, or doer of an action (e.g. the fireman), is placed pre-verbally (‘the fireman saved…’) and the patient, or object of the action (e.g. the baby), is placed post-verbally (‘…saved the baby’). They also have to learn that in passives, the word order of the thematic roles is reversed (‘the baby was saved by the fireman’).

Explaining when, and how, children master these different word orders is important for our understanding of the process by which children begin to be able to interpret the ambient language. For example, a consistent message in the literature is that active transitive structures are comprehended early by English-speaking children, even when novel verbs (such as ‘glorp’) are used to ensure that the task requires access to verb-general representations. Using preferential-looking, Gertner, Fisher and Eisengart [1] found that children as young as 1;9 can map basic active transitive sentences (e.g. the bunny is glorping the duck) onto a rudimentary form of sentential meaning, correctly interpreting the first noun phrase (NP) as the agent of the action and the second NP as the patient (see also [2] for German).

In stark contrast, the passive has traditionally been viewed as late acquired (e.g. [3–6]; see also [7]). Consistent with this view, research using act-out tasks has shown that even 3½ -year-old English-speaking children perform at chance levels with the passive, with both familiar and novel verbs (e.g. [8]). However, in studies using other experimental paradigms researchers have found earlier evidence of acquisition in English; structural priming studies (e.g. [9–10]) have demonstrated effects with the passive in English-speaking children as young as three years of age (though see [11] for a critique). The youngest age to date for which there is evidence that English-speaking children comprehend the passive structure with novel verbs is 2;10 [12].

### Frequency and the development of sentence processing heuristics

At least one of the factors contributing to the comparatively delayed acquisition of the English passive is its relative lack of frequency in child directed speech (e.g. [13]). Input frequency at the level of sentence frames like the passive is assumed to play a crucial role in syntax acquisition and processing in a number of theoretical models, including constructivism/ emergentism ([14–17]) as well as constraint-based approaches to sentence processing ([18]). However, another key factor may be the relative input frequency of an alternative sentence frame which competes for usage in that particular context (here: the active) (e.g. [19]). Because active transitive sentences are far more frequent than passive sentences in the input, English-speaking children are much more likely to hear sentences in which the first noun is an agent. When processing these types of sentences incrementally, they may develop a sentence processing heuristic in which they predict that the first noun is the agent. The development of such a frequency-based first-noun-as-agent heuristic is actually predicted by Chang, Dell and Bock’s [20] computational model of sentence frame processing and acquisition. The model learns its language representations by attempting to predict the input utterances in terms of message representations, which include semantic roles such as agent and patient ([21–22]). Since in English the agent maps onto the first noun more consistently than onto nouns in other sentential positions, the model develops stronger weights for these mappings early in development. When the ‘child’ version of the model is tested, these weights bias the model to exhibit a temporary on-line preference for an event which includes an agent at the timepoint during which the model is processing the first noun phrase. Over development, the model gradually learns to rely more on post-verbal structural cues to map sentences onto events. Because of this, it gradually unlearns its first-noun-as-agent preference. Of course, suggestions that English-speaking children might pass through a phase in which they use a first-NP-as-agent bias can be traced back to proposals by Bever ([23]) and Bates and MacWhinney ([24]). However, importantly these early researchers conceptualised this bias as one which (negatively) affected offline performance, (i.e. performance after the entire sentence has been processed).

### Incremental syntactic re-analysis: A developmental perspective

The distinction between an offline versus incremental first-NP-as-agent bias is an important one since adult listeners must reanalyse initial sentence mis-parses on a highly frequent basis during the most basic of conversations. Young primary-school aged children appear to find the process of syntactic reanalysis more challenging than do adults (e.g. [25–26]). For example, Trueswell, Sekerina, Hill and Logrip ([27]) found that if five-year-olds hear ‘put the frog on the napkin in the box’, they are much more likely than adults to fail to revise their initial interpretation of ‘on the napkin’ as the location into which they should place a frog, even though this interpretation becomes obviously incorrect once ‘in the box’ has been processed. Indeed, Kidd, Stewart and Serratrice ([28]) found that five-year-olds had great difficulty in comparison to adults in revising an initial mis-parse, even if this resulted in semantic implausibility (e.g. ‘cut the cake with a candle’, where candle is interpreted as the instrument).

### Consequences of incremental processing biases for English passive acquisition

These two sentence processing characteristics of English-speaking children (the incremental first-NP-as-agent bias together with developmental difficulties with syntactic reanalysis) would in combination have the following consequences for passive acquisition. First, they would influence how well children perform in comprehension experiments testing their knowledge of the passive. Even if children have already mastered the passive structure, they would be likely to mis-parse and thus misinterpret, passives in experimental situations. Second, these characteristics of the development of incremental processing may slow down the learning of passives; it would be difficult for children to learn how to use passive sentence structures if their sentence interpretation biases make it difficult for them to correctly interpret the passive sentences they hear.

However, we do not yet know whether young children have an incremental first-NP-as-agent bias. In children, the most well established evidence for a similar strategy comes from the types of errors that children make in sentence comprehension studies. For example, de Villiers and de Villiers [29] found that English-speaking two- to three-year old children pass through a stage in which they tend to act out passive sentences as if they were active transitives (i.e. as if the first NP were the agent; see also [5–6], [30]; as well [4], for similar findings using production). Relatedly, Gertner and Fisher [31] found that 21-month-olds showed an overall looking preference for a causative over a synchronous action when hearing intransitive sentences with two nouns as the subject (e.g. ‘The boy and the girl are glorping’). This suggests a tendency to assume that the first noun in a two-noun sentence is the agent of a causative transitive sentence.

However, none of these studies provide unambiguous evidence for an incremental first-NP-as-agent bias because all examined offline rather than incremental interpretations of the utterance. Because, in these paradigms, researchers only assess the children’s knowledge after the whole sentence has unfolded, there are alternative explanations for the results; including, for example, a bias to map the post-verbal noun onto the patient, or a bias to ‘count the nouns’ and map all sentences with two nouns onto causative meanings [32]. Alternatively, it could be that young children do not process at all the relevant morphological items (‘is’, ‘being’, ‘by’) which signal to the listener that these are not active transitive sentences. Indeed, it could be that young English-speaking two-year-olds have not even begun to distinguish the passive from the active frame.

It follows that the only way to conclusively determine whether young preschool children show an incremental first-NP-as-agent bias is to study how children’s sentence interpretation changes as the sentence unfolds in real time. The most sophisticated way of measuring incremental sentence processing is to use the ‘eye-tracking-while-listening’ method (e.g. [33]). Only one published study has examined online evidence for an incremental first-NP-as-agent bias in children. Huang, Zheng, Meng and Snedeker [34] carried out an eye-tracking study combined with an act-out measure. Five-year-old Mandarin-speaking children heard sentences in four conditions, whereby voice (active / passive) and the form of the first noun phrase (noun or pronoun) were fully crossed. The word order of all sentences was the same because voice was indicated by a marker (BA = active; BEI = passive) which occurred between the two noun phrases. Children heard a sentence (e.g. ‘the seal is being quickly eaten by it’ (lit: ‘seal BEI it quickly eat’) whilst viewing a ‘real’ visual world paradigm, namely three soft toys: 1) a plausible patient for the seal to act on (e.g. a fish), 2) a plausible agent who could act on the seal (e.g. a shark) and 3) the named referent (seal). In the act-out measure, the children were more likely to misinterpret the passives as active sentences in the noun-initial condition (‘seal BEI it quickly eat’) than in the pronoun-initial condition (‘it BEI seal quickly eat’). This was argued to indicate that in the noun-initial condition, children incrementally mapped the first encountered noun phrase onto the agent. Interestingly, both the eye-gaze measures and the act out data indicated that the children at least partially revised their initial first-NP-as-agent misanalysis. In the eye-gaze data, children distinguished active from passive sentences during the adverb (quickly) region. In the act-out data, if children had completely failed to revise their initial first-NP-as-agent analysis, then act-outs in the passive condition should have mirrored the active condition, which was not the case, indicating that these five-year-olds distinguished the two conditions.

Thus, in contrast to the frequent finding that five-year-olds have difficulties revising an initial syntactic analysis ([26,28]), these Mandarin-speaking children appear to be at least partially revising their initial misanalysis of noun-initial passive sentences. This raises the question of whether this holds for English-speaking children and indeed for younger children (and indeed whether children this age do in fact show an incremental first-NP-as-agent bias).

### The current study

The first aim of the present study was to determine whether English-speaking two- and three-year old children show a first-NP-as-agent bias in an online eye-tracking experiment. Since the visual world paradigm cannot be easily used with novel verbs, in Study 1 we used an eye-tracker in combination with an adaptation of the methodology of Gertner et al.’s [1] version of the novel verb preferential looking paradigm. We predicted that, when watching two simultaneous causative novel videos (one in which a boy is acting on a girl vs. one in which a girl is performing a different action on a boy), children will assume that the first NP is the agent and will thus look at the potential agent that corresponds to the first noun, before they have processed the rest of the sentence. Crucially, we also extended this paradigm to a condition containing passive sentences (e.g. ‘the boy is being glorped by the girl’) since, in a passive condition, an incremental first-NP-as-agent bias should lead to a temporarily erroneous interpretation (i.e. a garden-path, [25]). Because the time course of passive sentence interpretation is of key interest here, it was especially important to use novel verbs, since passive sentences with familiar verbs may in fact be more difficult for two-year-olds to interpret than passive sentences with novel verbs (e.g. [35] see also [36]). Thus, to investigate the first-NP-as-agent bias in both conditions we tracked eye movements after the onset of the first noun phrase.

Our second aim was to determine whether two- or three-year-old English-speaking children could recover from any mis-parse and demonstrate evidence of comprehension of the full passive with novel verbs. Although the literature is by no means unanimous (see e.g. [8, 11]), there are a number of indications that this should be achievable for 3½-year-olds ([9–10, 12]). However, no studies have tested children’s ability in an eye-tracking task. Crucially, no-one has examined passive sentence comprehension experimentally for children below 2;10 months [12], although we know eye-tracking can reveal sensitivity to sentence structure that is not observable in overt behavioural tasks (see [1]). Thus, the second aim of our study was to determine whether two-year-olds and three-year olds distinguished active from passive structures and moreover whether they correctly interpreted both sentence types overall.

To investigate this second question, we used two measures. First, in Study 1, in a second analysis, we time-locked eye movements to the onset of the second noun phrase of both active and passive sentences, where the two became structurally equivalent. Second, in Study 2, we used a pointing paradigm with similar materials and a new sample of children to determine whether any discrimination between the active and passive sentence frames found using eye-tracking measures would be replicated when children this age are asked to make a behavioural forced choice.

Thus, in sum, in the current paper we asked:

1. Is there is an incremental first-NP-as-agent bias in young English-speaking pre-schoolers? (Study 1: Analysis 1)
2. Are young preschool children able to partially revise an initial syntactic mis-parse? That is, do either two- or three-year-olds recover from an incremental first-NP-as-agent bias to then distinguish active from passive sentences? (Study 1: Analysis 2 and Study 2)

## Study 1: Preferential looking with eye-tracking

### Method

#### Participants

We tested typical-developing, monolingual British English-speaking children in the Kent Child Development Unit. This study was approved by the University of Kent UK Ethics Committee. Informed written consent was obtained from the caregivers, and the children gave verbal consent. There were two age groups: fifty-six 2-year-olds (M = 25.4 months, range = 23–28 months, 32 boys; 28 in each condition); fifty-eight 3-year-olds (M = 41.82, range 39–44 months, 30 boys; 29 in each condition). All were monolingual and typically-developing. We also tested an additional 11 participants, but these were excluded due to bilingualism (1), diagnosis of a developmental disorder (2), calibration error (2), other technical errors (2) or more than half the data being lost during the test trials (4). The 2-year-old Active and Passive groups did not differ on the receptive vocabulary sub-test of the Wechsler Preschool and Primary Scale of Intelligence 3 (WPPSI)[37], (Active: M = 10.61, SD = 6.02; Passive: M = 10.33, SD = 4.92, p = .86) nor on the Lincoln Toddler CDI Vocabulary Comprehension ([38]; Active: M = 514.12, SD: 152.62; Passive: M comprehension score = 528.00, SD = 144.00, p = .74) nor on the Lincoln CDI Vocabulary Production (Active M = 367.16, SD = 186.98, Passive M = 383.42, SD = 178.70,, p = .75). (The WPPSI and CDI measures were strongly and positively correlated for the two-year-olds, all r < .5 and all p < .001).

The 41-month-old Active and Passive groups did not differ on mean raw scores on the WPPSI receptive vocabulary (Active M = 19.79, SD = 5.33, Passive M = 21.00, SD = 4.33, p = .35), Clinical Evaluation of Language Fundamentals- Preschool [39] expressive vocabulary (Active M = 19.68, SD = 6.18; Passive M = 19.48, SD = 4.20, p = .89) or Communicative Development Inventory (CDI) III [40] vocabulary production scores (Active M = 78.38, SD = 14.92; Passive M = 77.36, SD = 17.12, p = .82). (The WPPSI and CELF were moderately-strongly correlated (r(57) = .469, p < .001. The CELF and CDI III were moderately correlated (r(53) = .275, p < .05). The WPPSI and CDI III were not correlated, p = .301).

#### Design

Age Group (25-month-olds vs. 41-month-olds) and Sentence Structure (Active vs. Passive) were both between-subject variables. The dependent variable was the mean proportion of looks (in 20 msec time bins) to the video that matched the active transitive interpretation of the test sentence (active match), which was the target for the active condition and the non-target for the passive.

#### Materials

To adapt Gertner et al.’s [1] study 3, we created twelve 8000 msec long videos based on novel actions drawn from Gertner et. al. [1], Noble, Rowland and Pine [41] and Naigles [42]. Each used girl and boy characters, as in Gertner et al.’s [1] third study. Each action was causative in that an agent acted in a way that caused a movement or change of location in the other character. The 12 novel actions we used had the highest agent-caused-action ratings out of a sample of 20 that were rated by naive adults. (Adults were asked to rate on a seven-point Likert scale the degree to which the action was ‘caused’ by the girl versus the boy). Thus, in our study, for each test trial, we paired novel action videos on the basis of these causality ratings, such that each member of a pair had a similar causality rating to the other (see S1 Appendix for pairings).

One key modification that we made to the test trials (novel verb trials) component of Gertner et al.’s [1] paradigm was that in our study each test sentence was only heard once and only in the present tense. Another key modification we made to the test trials was that instead of first showing the participants each novel event separately (in familiarisation trials), we instead first showed the participants a silent version of each video clip pair, which ran for 8000 msec without any audio stimulus. Since the causative actions were iterative and durative, we looped the original clips so that the same actions continued for another 8000 msec after the onset of the audio stimuli. We also replaced the Gertner et al’s [1] initial character identification trials with two object-recognition trials based on Fernald, Pinto, Swingley, Weinberg and McRoberts, [43], and added extra object recognition trials after the last test trial. This was because in an earlier version of this paper we used the data from these object-recognition trials to calculate the mean latency (over each age group) to switch from the distracter object to the target object at the word onset. This mean lexical processing speed was then factored into analyses (at the age group level) using pre-determined 800 msec time regions. The results using this earlier analysis were very similar to those presented in the current study; both age groups showed an incremental bias to map the first NP onto the agent and both age groups differentiated the Active from the Passive structure after the second NP. However, there were many problematic assumptions with regards to determining the offset for processing speed. First, there are large individual differences in speed of processing within an age group. This is not unsurmountable per se but in fact processing speed also appears to vary as a function of the precise stimuli used (cf. [43] with [44]); it is unclear that the lexical processing speed required for a switch task like Fernald et al.’s [43] would be equivalent to the lexical processing speed required in our task. In addition, it is unclear how to define time windows for the differentiation between Active and Passive in children this young. Therefore, the permutation analysis presented below is ultimately more satisfactory since it requires fewer assumptions.

Since our participants were learning British English in which the passive is typically used with ‘is’ rather than ‘get’ as the auxiliary (e.g. [45]), our test sentences had the form ‘X is being VERBED by Y’, as shown in S2 Appendix. The audio stimuli were recorded by a female, native speaker of British English and were edited using Wavepad. The same two sentence-initial ‘ambiguous’ phrases (‘the girl is....’ for half the sentences and ‘the boy is…’ for the other half) were spliced into the beginning of each sentence. The mean total sentence length for the Active test sentences was 3610 msec and for the Passive 4087 msec (see S2 Appendix for test sentence stimuli set). The Passive counterparts to the Active sentences started with the same initial phrase so that the Active and Passive sentences of each particular counterbalanced order were identical until the offset of ‘is’.

#### Procedure

Children were seated in a car seat 70 cm in front of a 19” computer screen and a Tobii X120 eye-tracker and were instructed to keep their hands in their laps and not to point. The experiment began with a nine-point calibration, followed by four phases:

Phase 1: Two object-recognition trials.
Phase 2: Two Real Verb trials. Here, the target clip of each pair could be identified both by the action (e.g. washing vs. feeding) and by the syntax. For these Real Verb trials, participants did initially see (following Gertner et al. [1]) familiarisation clips, where they viewed the two dynamic video clips sequentially, one after the other, along with the audio stimulus ‘Look!’. Then, participants heard the test sentence in the future tense whilst watching a black screen. This was followed by the two dynamic clips side by side for 8000 msec, whilst the participants heard the test sentence in the present tense (e.g. ‘the boy is being washed by the girl’). Then, participants heard the test sentence in the past tense whilst watching a black screen, followed by a Tobii attention getter designed for infants, which moved and made a noise in the centre of the screen for three seconds prior to each trial. Then, participants heard one more repetition of the audio stimuli accompanied by the video clips for 16000 msecs (e.g. ‘the boy is being washed by the girl. Find washing’), with the audio starting at 8000 msec.
Phase 3: Six novel verb trials. Here the participants only heard each test sentence once and only in the present tense. As in Gertner et al.’s [1] study, for each test trial, each child saw two simultaneous novel actions, each involving a girl and a boy. The semantic roles (agent / patient) of one video clip (e.g. girl swivels boy in chair) were the reverse of that of the other video clip (e.g. boy pushes girl back and forth on trolley). Children in the Active condition heard for example ‘the boy is pogging the girl’ whereas children in the Passive condition heard for example ‘the boy is being pogged by the girl’. There were no familiarization clips. Rather, after the Tobii attention-getter, participants saw the two clips side by side playing for 16000 msec, with the audio stimulus (e.g. ‘the boy is being pogged by the girl. Find pogging’) starting at 8000 msec.
Phase 4: Eight further object recognition trials. These are not presented in the analysis below.

#### Counterbalancing

The test trials were counterbalanced between-subjects for a) the sentences heard (A vs. B list, see S2 Appendix); b) which event of each pair was the target; c) the order in which the novel verbs and actions occurred and d) the order for sides of the screen on which the target appeared. We also ensured that the following were counterbalanced both between and within participants: whether the girl or the boy was the agent of the target action and which side of the screen was the target. The familiar verb trials were counterbalanced in the same way.

### Analysis

#### Gaze-data processing

Each novel verb test sentence was heard only once (in the present tense) and there were six test trials. Eye movements that were initiated while the test videos were onscreen were time-locked to key onsets of audio stimuli (the onset of the first NP or the onset of the second NP, see the Results section for more details). The spatial coordinates of fixations (in pixels) were mapped onto two areas of interest, corresponding to the dimension of each video clip, one on the left and one on the right hand side of the screen (A on the left, and B the same size on the right side of the screen) where the two videos simultaneously appeared. For each trial, either A or B showed the video clip in which the first-mentioned NP was the agent (active match), and this was counterbalanced both within and across participants. Data were collated via Matlab into 20 msec time-bins, where fixations to the video that matched the active sentence (active match) were coded 1 and fixations to the video that matched the passive sentence (passive match) were coded 0. Any data that were deemed invalid due to blinks or head movements, or where no fixations to either region were recorded, were removed from the dataset. Thus, looks to the active-match versus looks to the passive-match were essentially in complementary distribution.

#### Permutation analysis

In adult eye-tracking studies, one can estimate the time between stimulus and related eye-movements, and it is also possible to develop windows of analysis based on previous studies. In contrast, when eye-tracking young children, it is not clear how much time is required to process the stimuli and there may not be sufficient data for estimating appropriate time windows (cf. [43] with [44]). Therefore, our data were analysed using a permutation test technique that was originally developed for use with time-course data, such as ERP data, for which it is difficult to know a priori which time regions are relevant and which statistical corrections for multiple comparisons are required ([46]; see [47– 49] for use with the eye-tracking-while-listening paradigm with children). The first step involved computing the test statistic on the real data for each possible time point (in our case the 20-msec time-bins created via Matlab). For the second step, adjacent time-bins for which the test statistic was significant at the 0.05 level were clustered together. This captures the assumption that similar differences in adjacent time points are likely the result of a single processing component, which is represented by the cluster. We then generated a distribution by permuting condition labels in our data (e.g., active, passive) and this created an exact distribution specifying how likely the data that we did collect would have occurred by chance if we carried out the experiment multiple times and permutated the labels. We then compared our cluster statistic against this distribution to determine the significance of our effects.

In some eye-tracking studies, individual comparisons are applied to individual windows even when an omnibus condition by window interaction has not been found. This inflates the chance of obtaining an effect since multiple comparisons are carried out across the windows. To address this problem, we compared each of the clusters that were identified in the clustering step against a permutation distribution that had the largest test statistic for each permutation. In generating the permutation distribution, we took the original eye-tracking data and permuted the labels (that is, we randomly mixed active/passive labels). Each permutation is akin to a new experiment with 114 children, except that here the labels are randomly linked to the data points from the original dataset. Using all of the clusters generated from the actual data, we computed a sum test statistic for each cluster in this simulated experiment. Then we selected the maximal sum test statistic for this experiment and included that in the permutation distribution ([46]). If we did not select the maximal statistic, then the smaller clusters would contribute to the centre of the distribution and that would make it easier to find a significant effect (see [49] for an illustration of this point and more information about the analysis). The permutation distribution included the maximal sum test statistics across a large number of experiments (e.g., 1000). We computed p-values for particular clusters by computing the proportion of values in the distribution that were greater than the cluster test statistic. If less than 2.5% of the distribution was greater than the test statistic, then it was significant by a two-tailed test. While degrees of freedom are used to map ideal distributions (e.g., F) to particular data sets, our permutation distribution is an exact distribution for the number of participants in this study, and hence degrees of freedom are not needed. Since all of our clusters were compared against a permutation distribution that was generated from the maximal test statistic across the multiple clusters that we were using, our distribution was designed to test for significance across the actual number of comparisons that we were performing.

## Results

### Do young children show a first-NP-as-agent bias?

**Fig 1:**
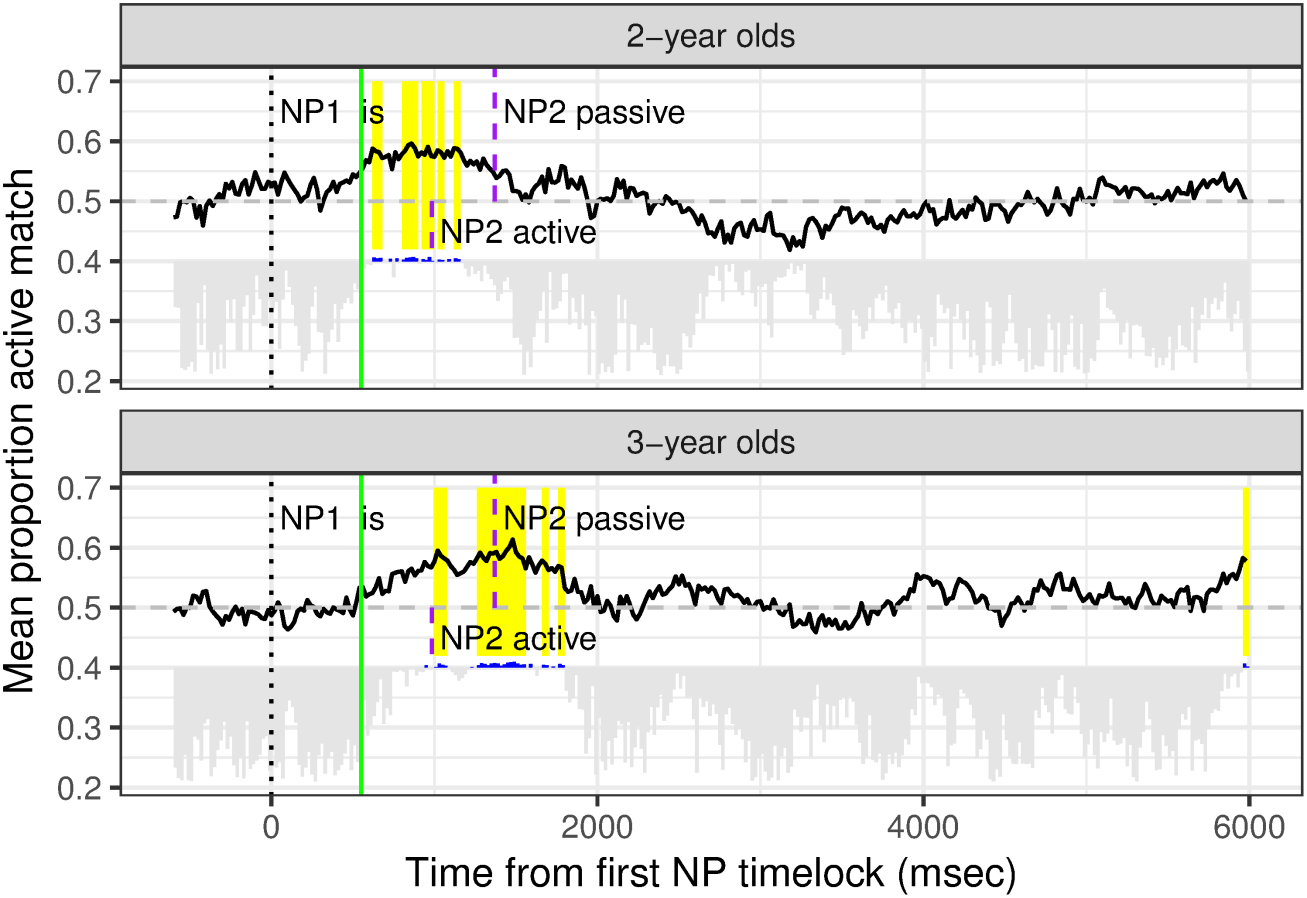
Significant clusters (yellow) for mean proportion looks to the clip in which the first mentioned NP is the agent. 0 indicates the onset of the first NP. Grey bars extending below the horizontal line indicate 20msec timebins for which the first-NP-as-agent-bias is not significant; the longer the bar, the further from significance. Significant timebins are indicated by a blue indentation above the horizontal line which is positioned at 0.4.

In order to investigate whether young children show a bias to map the first NP incrementally onto the agent of a causative action, in our first analysis we used Matlab to time-lock to the onset of the first NP; i.e. the capitalized word in, for example, (active condition) ‘THE boy is keefing the girl’ and (passive condition) ‘THE boy is being keefed by the girl’. Data from each 20 msec timebin were aggregated over the 6 test trials to yield an overall proportion active match score. Since this analysis focuses on examining whether there is an incremental bias before the children have processed the morpho-syntactic differences between the active and the passive, Fig 1 shows the proportion active match averaged across Structure. A score above 0.5 reflects a preference to fixate on the video clip in which the first mentioned NP was the agent.

To determine whether either age group showed a first-NP-as-agent bias, we carried out a permutation analysis. The first step in this analysis is to compute a test statistic for each individual 20 msec timebin. We applied a linear regression with a centred predictor variable for Structure (Active vs. Passive) to the data from each timebin. The proportion active match was transformed with the empirical logit to map onto a continuous range. The intercept for the regression provides a t-test for whether the logit active match is greater than 0, which is equivalent to whether the proportion active match is greater than 0.5. If the intercept is significant and positive, that would provide evidence for a first-NP-as-agent bias across children in a particular timebin. The results of the first step are shown as bars that are placed arbitrarily at 0.4 on Fig 1. That is, if the grey bar extends below this line, the p value for the intercept is greater than 0.05 for this 20 msec timebin. (The length of the bar that extends below the line is the exact p value subtracted from 0.05; thus, the longer the bar, the further from significance). If there is no bar extending below the line, but instead there is a blue indentation above this horizontal line, then the intercept was significant for this 20 msec timebin.

The second step is to cluster adjacent timebins together if they have significant intercept p-values for the intercept in the first step. When the blue bars in Fig 1 are connected together, that represents a single cluster. This process identified 8 clusters in the 25-month-olds and 9 clusters in the 41-month-olds. We then created a permutation distribution to test whether these clusters are significant. This was done by taking the data for each 20 ms timebin and permutating the order of the structure labels and fitting a similar regression model to the data. The intercepts for these simulated models were stored and used to create a sum test statistic for each cluster; that is, the sum of the intercept t-values for each cluster. Following this, for each simulation, the maximal sum test statistic was identified and this was included in the permutation distribution for each age group. Then the sum test statistic for the clusters that were found in the actual human data were computed and compared against the permutation distribution for their age group. The p-value was the proportion of the permutation distribution that was greater than the cluster sum test statistic. If the p-value was less than 0.025 (significant by a two-tailed test), then we can conclude that the observed results are unlikely to occur by chance given the variability in this population and the number of comparisons tested.

The clusters that are significant by the permutation distribution are highlighted in Fig 1 by the yellow overlaid regions. We found that the 25-month-olds looked more at the first-NP-as-agent scene in multiple clusters (620–680 msec, 800–900 msec, 920–1000 msec, 1020–1060 msec, 1120–1160 msec). The earliest cluster began 620 msec after the onset of the first NP, which is 362 msec prior to the onset of the 2^nd^ NP. Interestingly, this significant cluster occurred only 69 msec after the onset of the verb morphology ‘VERBing’ (at 551 msec). Given that previous studies with children this age estimate about 750 msec lexical processing time (e.g. [50–51]), it is unlikely that there is sufficient time to process even the lexical information in the verb phrase, let alone the structural information. Thus, we conclude that this processing component is based on the information in the first noun phrase and indicate a first-NP-as-agent bias.

The 41-month-olds also looked at the first-NP-as-agent scene in multiple clusters (1000–1080, 1260–1560, 1660–1700, 1760–1800, and 5960–6000 msecs). The last cluster starting at 5960 is very long after the whole sentence has been processed and thus is not related to the processing of the first NP. The earliest cluster began at 1000 msec after the onset of the first NP, which is prior to the onset of the second NP in the Passive condition (at 1370) and only 17 msec after the onset of the second NP in the Active condition (at 983 msec), which, again, is clearly too soon for the second NP to have been processed. Importantly, for both age groups, the trend towards this particular cluster clearly started well before the onset of the second NP.

### Do two and three-year-old children discriminate active from passive sentences?

Our second analysis tested whether there was any evidence that children differentiate Active from Passive sentences. Since the Active and Passive conditions differed from one another both in the length and type of morphology from the offset of the verb phrase, which is a problem for statistical comparison of time-windows across active and passive structures, we time-locked the data to the onset of the second NP, after which point Active and Passive sentences were identical

**Fig 2:**
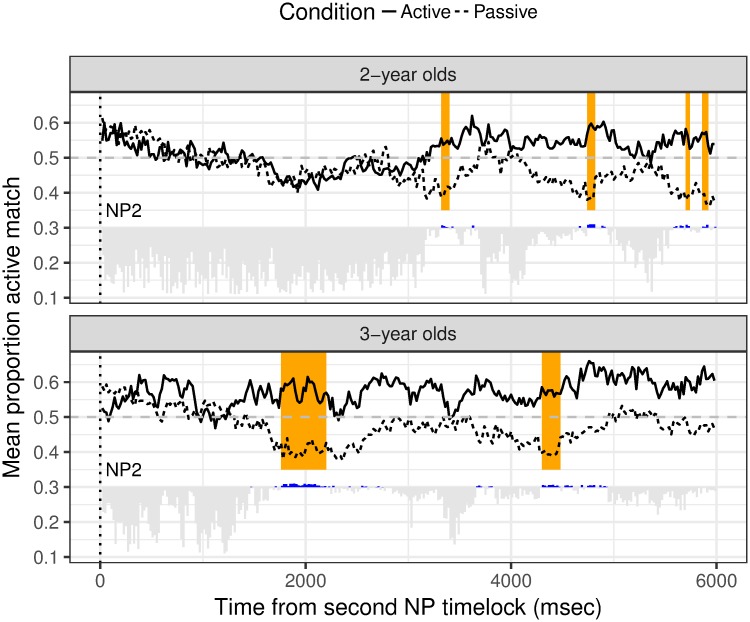
Significance windows for discrimination of active from passive structure by age. 0 indicates the onset of the second NP. Grey bars extending below the horizontal line 20msec timebins for which the first-NP-as-agent-bias is not significant; the longer the bar, the further from significance. Significant timebins are indicated by a blue indentation above the horizontal line.

Eye-movements that were initiated while the test videos were on screen were again time-locked in Matlab on a trial-by-trial basis, but this time to the onset of ‘the’ in the second NP; i.e. the capitalised word in for example (active condition) ‘the boy is keefing THE girl’ and (passive condition) ‘the boy is being keefed by THE girl’. Data from each 20 msec timebins were aggregated over the 6 test trials to yield an overall proportion active match score. Since this analysis examines the difference in the way the structures were processed, we show the proportion active match separately for the Active and Passive structures in Fig 2. To determine whether the children discriminated between the structures, we carried out a second permutation analysis. As before, we used a linear regression to predict empirically transformed logit active match using a centred predictor variable for Structure (Active vs. Passive) for each timebin. The t-test for Structure provides evidence for the ability to distinguish structures at each timebin and we show this t-value arbitrarily at 0.3 in Fig 2 (blue bars indicate significance at the 0.05 level). After clustering adjacent windows together based on the significance of Structure (Active / Passive), we identified 11 clusters in the 25-month-olds and 16 clusters in the 41-month-olds. We then created a permutation distribution using the same process as for the first-noun-as-agent, but here using the t-statistic for Structure (Active / Passive) as our measure. For each simulation, the maximal sum test statistic for Structure was identified and included in the appropriate age-specific permutation distribution. Then the sum test statistic for the clusters that were found in the actual human data was computed and p-values were computed by looking at the proportion of permutation distribution values that were greater than the observed sum test statistic for each cluster.

The 41-month-olds distinguished between Actives and Passives in two clusters, the first of which extended from 1760 msec to 2200 msec after the onset of the second NP and the second between 4300 and 4480 msec. The 25-month-olds showed a significant difference in looking behaviour between Actives and Passives in multiple clusters (3320–3400, 4740–4820, 5700–5740 and 5860–5920 msec). The divergence is—as for the 41-month-olds—in the target direction for each audio condition but for the 25-months each cluster was very brief in duration.

## Study 2: Active and passive comprehension using forced choice pointing

### Method

#### Participants

A different set of participants took part in the pointing study. This study was also approved by the University of Kent Ethics Committee. Informed written consent was obtained from the caregivers, and the children gave verbal consent. We tested typical, monolingual British English-speaking participants in two age groups: 25-month-olds and 41-month-olds. All participants were tested in the Kent Child Development Unit. Twenty-four 25-month-olds were assigned to the Active Structure condition (M = 26.08 months, range 23–28 months, 11 boys) and 27 to the Passive (M = 25.85 months, range 23–28 months, 11 boys). The groups did not differ in expressive vocabulary as measured by the SprachEntwicklungsTest-Kindergarten-2 (SETK-2 [52] English translation; Active M = raw score of 19.24, SD = 8.71, Passive M = 21.15, SD = 4.43, p = .410). (One 2-year-old in the Passive condition and seven in the Active condition were not administered this test. Instead they were administered the Expressive One-Word Test [53] which was found to be too long in duration for this age group). Twenty-six 41-month-olds were assigned to the Active Structure (mean age = 41.23 months, range 39–44, 14 boys) and 24 to the Passive (mean age = 40.92 months, range = 39–44, 11 boys). The groups did not differ in expressive vocabulary as measured by the CELF-P [39]: Active M raw score = 19.58, SD = 5.29, Passive M raw score = 17.08, SD = 6.33, p = .136. The three-year-olds also did not differ regarding vocabulary scores on the CDI III [40]: Active M = 72.96 (SD = 20.62), Passive M = 80.1 (SD = 10.44), p < .145. (Parents of five included three-year-olds did not wish to complete this questionnaire). The CELF and CDI III were moderately correlated (r(45) = .330, p < .05). An additional 25 children were tested but removed from analyses because of experimenter error (1), because all their test trials were uncodable, because, for example, the touch-screen did not record the response (four, all of whom were 25-month-olds), or because they showed a side bias (sixteen 25-month-olds, four 41-month-olds), defined as pointing to the same side for all of the six test trials. Thus we excluded 28% of our 25-month-olds. This rate is similar to other studies that have found above-chance performance for English-speaking two-year-olds with novel verbs heard in the active transitive using this task; Dittmar, Abbot-Smith, Lieven and Tomasello [54] excluded 44% of 25-month-olds for side bias or fussiness and Fernandes, Marcus, deNubila and Vouloumanos [55] had the same exclusion rate for 30-month-olds. Our exclusion rate for 41-month-olds was 7%.

#### Design

The study employed a between-subjects design with two IVs; Age (25-vs. 41-month-olds) and Sentence structure (Active vs. Passive). The dependent variable was the same as for the eye-tracking study (Study 1); that is, the proportion of points that the participants made to the video which matched an active interpretation (active match).

#### Procedure

The procedure was based on a forced-choice pointing task developed by Noble et al. [41], which they used to test how 27-month-olds comprehended the active transitive with novel verbs. The participants were told that they were going to see two videos on the screen, but that Piglet (a hand-puppet with a loudspeaker inside) was only going to talk about one of them. Their task was to touch the video that Piglet was talking about. The experiment had three phases following Noble et al.’s [41] procedure:

Phase 1: Three Intransitive Novel Verb Training trials. The children heard novel verbs in an intransitive sentence (e.g. ‘the girl is raxing’) and were asked to choose between a video of a girl standing still and the same actress moving in a novel way on the other side of the screen.
Phase 2: Two Real Verb Practice trials. Children first saw two still clips side-by-side. In each, there were two characters (boy and girl) performing a familiar causal action. Participants then saw a blank screen and heard the test sentence in the future tense (e.g. ‘the boy is going to be washed by the girl. Find washing’). Then participants again saw each clip side-by-side but this time in dynamic motion (for 8 seconds) accompanied by the test sentence in the present tense (e.g. ‘the boy is being washed by the girl. Find washing’). The target and foil clip depicted two different familiar actions (e.g. ‘washing’ pitted against ‘feeding’) and were also differentiated by which character was the agent (e.g. in this instance the boy character was the patient in the washing clip but the agent in the feeding clip). Children in the Passive condition heard audio stimuli in the passive sentence frame only and children in the Active only in the active frame. Feedback was given for both the Intransitive Novel Verb Training trials and Real Verb trials.
Phase 3: Six test trials. There were six test trials with no feedback, but trials could be re-run once if the child did not respond the first time. Responses were recorded by a touch-screen. For the test trials, participants saw two still clips side-by-side with the same two characters in each, but engaged in two different novel actions. Participants then saw a blank screen and heard the test sentence in the future tense (e.g. ‘Look! The girl is going to be semmed by the boy’). Then participants again saw the pair of clips (target and foil) side-by-side, but this time in dynamic motion (for 8 seconds) accompanied by the test sentence in the present tense (e.g. ‘The girl is being semmed by the boy. Find semming!’). The experimenter also asked the child to e.g. ‘Touch …/ Point to where … the girl is being semmed by the boy’.

#### Materials

The audio-visual materials for the Real Verb and Test (novel verb) Trials were the same as for Study 1 except for the changes necessary to adapt these to Noble et al’s [41] procedure as described above. Counterbalancing was as for Study 1.

#### Results and discussion

**Fig 3:**
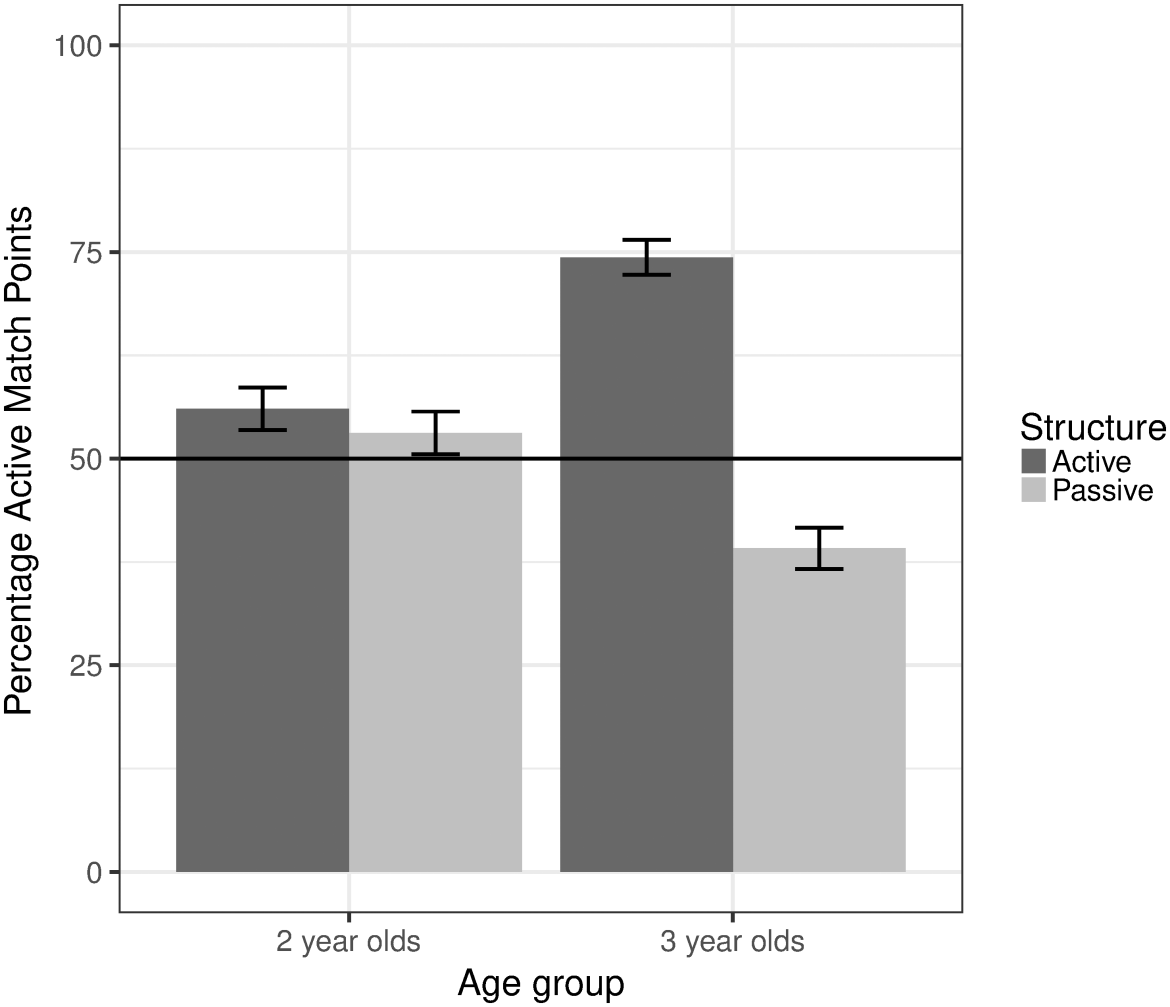
Mean percentage active match points by age and structure.

The key aim of Study 2 was to determine whether English-speaking 25- and 41-month-olds would distinguish the full passive from the active using novel verbs in an offline task. Fig 3 thus shows for each Sentence Structure (Active vs. Passive) and Age (25-vs. 41-month-olds) the mean percentage of points to the video which matched an active interpretation (active match). Thus, if a group had performed at ceiling in terms of accurate comprehension, the dependent variable would have been 100% in the Active condition and 0% in the Passive condition. We used binominal mixed effect models with effect coded factors (e.g., [56]), whereby the factors were Sentence Structure and Age, fully crossed. Participants and items were entered as random effects with by-item slopes for Structure. P-values were computed by comparing models with likelihood-ratio tests and chi-square values are reported. There was a main effect for Structure indicating (as expected) that overall participants pointed to the active-match video less often for the Passive than for the Active Structure (b = -0.85, SE = 0.21, χ^2^(1) = 8.67, p < .01). There was no main effect for Age (p = .63). However, this needs to be interpreted in the light of an interaction between Structure and Age (b = -1.45, SE = 0.39, χ^2^(1) = 13.91, p < 0.001). To follow up this interaction, we fitted separate models for each Age with participants and items entered as random effects with by-item slopes for Structure. There was no significant effect of Structure for the 25-month-olds (p = .66), indicating that this age group did not distinguish the two structures in this task. However, the 41-month-olds pointed more frequently to the active-matching video in the Active than in the Passive condition (b = -1.60, SE = 0.30, χ^2^(1) = 15.96, p < .001), clearly indicating that they distinguished the two structures in this forced-choice task as well as in the eye-tracking task.

Thus, in Study 2, 41-month-olds successfully demonstrated that in pointing, as for eye-tracking, they discriminated active sentences from full passive transitive sentence frames containing novel verbs. The 25-month olds did not discriminate the actives from the passives in the pointing task.

## General discussion

We investigated two questions relating to how English-speaking preschool children (at 25 and 41 months) interpret active transitive and passive sentences. First, we eye-tracked them to investigate whether they show an incremental first-NP-as-agent-bias; that is, a bias to map the first noun of the sentence onto the agent of a causative event, before the remainder of the sentence has been processed. Second, we used eye-tracking (Study 1) and pointing (Study 2) measures to investigate whether children at this age discriminate active from passive sentences, ultimately mapping the first noun of passive sentences onto the patient of a causative event.

Following recent developments within the eye-tracking-while-listening paradigm ([47–49]), we used permutation analysis to analyse the eye-tracking data. This allows us to avoid pre-specifying the analysis windows whilst simultaneously correcting for multiple comparisons (see [46]), and to avoid making assumptions regarding by how much we should offset our data analyses to allow for differences in processing speed—a particular problem when comparing across developmental groups.

To investigate whether 25- and 41-month-olds showed an incremental first-NP-as-agent bias, our first analysis timelocked to the onset of the first NP; we examined which of two novel causative events children looked at before they had processed the second noun phrase of the sentence. We found that both age groups showed this incremental bias. For the 25-month-olds, the earliest cluster indicating a first-NP-as-agent bias commenced at 620 msec after the onset of the first NP, well before the onset of the second NP, and also prior to the time-point at which the verb morphology could plausibly have been processed. The 41-month-olds also showed an incremental first-NP-as-agent bias, the initial incline of which started around the same time as that for 25-month-olds.

To investigate whether children distinguish active from passive sentences we used two measures. In Study 1, we again examined eye-gaze preference, but here we time-locked to the onset of the second noun phrase. In Study 2, we used a forced-choice pointing measure. Both measures show that 41-month-old children clearly distinguish active transitive from full passive sentences. Moreover, for both structures, the means are in the target direction; that is, once the whole sentence has been processed the 41-month-olds (at the group level) mapped the passive sentences onto the correct video. However, the results for the 25-month-olds are less clear. In Study 1, the response of 25-month-olds to active and passive sentences also seems to diverge after the onset of the disambiguating material, and again the means are in the target direction. However, the clusters which reached significance were only very brief in duration which suggests that the timing of looks to the correct video for each structure was variable across the younger children. The first of these clusters only occured around 3320 after the onset of the second NP, which suggests that their knowledge of the structural distinction is still fairly weak. Moreover, the forced-choice pointing data show no evidence that our group of English-speaking 25-month-olds distinguish active from passive sentences.

While our finding for active-passive discrimination in 25-month-olds is certainly not robust and needs further exploration and replication in future studies, our finding of an incremental first-NP-as-agent bias in this age group fits with previous offline findings for English-speaking preschoolers ([29, 31, 57]). That is, if two-year-olds find it difficult to revise an initial mis-parse / garden-path, then an incremental first-NP-as-agent bias would also manifest itself as an offline whole-sentence first-NP-as-agent bias. This would mean that passive sentences would systematically be interpreted as active transitive sentences. Of course, an incremental bias of this type is unlikely to be the only reason why very young children might interpret passive sentences as active ones. Likely contributing factors include the overall frequency of the passive in a particular language (e.g. [58]), its degree of semantic restriction (e.g. [59–60]) and the degree to which children have acquired the relevant components of passive morphology such as (for English) the auxiliary ‘be’, the participle and ‘by’ (e.g. [19]).

In contrast to our findings for 25-month-olds, our findings for the 41-month-olds are clear. These older pre-schoolers also showed an incremental first-NP-as-agent bias. Unlike the 25-month-olds, however, the 41-month-olds were clearly able to revise this initial ‘garden-path’ incremental parse to map passive sentences onto the target meaning most of the time. This ties in with previous findings that English-speaking three-year-olds are able to access a verb-general representation of the full passive (e.g. [9–10], [12]). Importantly, this also indicates that an incremental first-NP-as-agent bias will not in itself prevent a target-like offline interpretation of a passive sentence. Rather, such a bias will only hinder passive interpretation if accompanied by difficulties with syntactic revision. Indeed, our finding that three-year-olds can revise an initial incremental mis-parse of passive sentences extends the findings of Huang et al. [34], who argue this point for their sample of Mandarin-speaking five-year-olds.

That said, it is, of course, possible, as argued by Kidd et al. ([28]), among others, that young children only partially revise initial sentential mis-parses (garden paths) and that syntactic revision is more difficult for children than for adults, perhaps because of the burden on executive functioning. This may in part explain why, despite evidence of competence with the English passive by three years, children’s performance with the passive has frequently shown a protracted period of error-prone comprehension and usage ([59, 61]). Nonetheless, the process of syntactic revision in adults is certainly not error-free. Indeed, there is some evidence that even after adults have revised an initial garden-path, the initial mis-parse lingers in some form to interfere with offline sentential interpretation ([62]). Therefore, it would not be surprising if children’s syntactic revision is ‘partial’ in a similar sense, although clearly further research is needed to investigate the developmental trajectory of these revision strategies.

### Conclusion

In sum, the current study is the first to demonstrate that young English-speaking preschool children show a bias to incrementally map the first noun in a sentence onto an agent role. Nevertheless, English-speaking three-year olds can revise this initial mis-parse, to distinguish between active and passive sentences and map passive sentence frames onto the appropriate sentential meaning. The results are consistent with computational models of language acquisition in which distinct incremental form-meaning biases for the first noun phrase versus other structural cues appear early in development if supported by characteristics of the input.

## Supporting information

S1 Appendix Visual stimuli: Video clip pairs and their associated novel verbs used in the test trials of both eye-tracking and pointing tasks. (DOCX)

S2 Appendix Sentence stimuli. (NB: Order of verb-action pairs was counterbalanced according to Latin squares. Therefore only 1/8 children started with mabbing).
(DOCX)

## References

[1] Gertner Y, Fisher C, Eisengart J. Learning words and rules: Abstract knowledge of word order in early sentence comprehension. Psychol Sci. 2006 8; 17(8): 684–691. doi: 10.1111/j.1467-9280.2006.01767.x 16913951 10.1111/j.1467-9280.2006.01767.x

[2] Dittmar M, Abbot-Smith K, Lieven EVM, Tomasello M. Young German children’s early syntactic competence: a preferential looking study. Dev Sci; 2008 6; 11(4): 575–582. doi: 10.1111/j.1467-7687.2008.00703.x 18576965 10.1111/j.1467-7687.2008.00703.x

[3] Baldie BJ. The acquisition of the passive voice. J Child Lang; 1976 10; 3(3): 331–348. doi: 10.1017/S0305000900007224

[4] Brooks PJ, Tomasello M. Young children learn to produce passives with nonce verbs. Dev Psychol. 1999; 35(1): 29–44. doi: 10.1037/0012-1649.35.1.29 9923462 10.1037//0012-1649.35.1.29

[5] Horgan D. The development of the full passive. J Child Lang. 1978 9; 5(1): 65–80. doi: 10.1017/S030500090000194X

[6] Lempert H. Animacy constraints on preschool children’s acquisition of syntax. Child Dev. 1989 2; 60(1): 237–245. doi: 10.2307/1131088

[7] Armon-Lotem S, Haman E, de Lopez K, Smoczynska M, Yatsushiro K, Szczerbinski M. et al A large-scale cross-linguistic investigation of the acquisition of the passive. Lang Acquis. 2016; 23(1): 27–56. doi: 10.1080/10489223.2015.1047095

[8] Pinker S, Lebeaux DS, Frost LA. Productivity and constraints in the acquisition of the passive. Cognition. 1987 8; 26(3): 195–267. doi: 10.1016/S0010-0277(87)80001-X 3677572 10.1016/s0010-0277(87)80001-x

[9] Bencini GML, Valian VV. Abstract sentence representations in 3-year-olds: Evidence from language production and comprehension. J Mem Lang. 2008 7; 59(1): 97–113. doi: 10.1016/j.jml.2007.12.007

[10] Shimpi P, Gamez P, Huttenlocher J, Vasilyeva M. Syntactic priming in 3- and 4-year-old children: evidence for abstract representations of transitive and dative forms. Dev Psychol. 2007; 43(6): 1334–1346. doi: 10.1037/0012-1649.43.6.1334 18020815 10.1037/0012-1649.43.6.1334

[11] Kidd E. Individual differences in syntactic priming in language acquisition. Appl Psycholinguist. 2012 4; 33: 393–418. doi: 10.1017/S0142716411000415

[12] Ibbotson P, Theakston A, Lieven EVM, Tomasello M. The role of pronoun frames in early comprehension of transitive constructions in English. Lang Learn Dev. 2011; 7(24): 24–39. doi: 10.1080/15475441003732914

[13] Gordon P, Chafetz J. Verb-based versus class-based accounts of actionality effects in children’s comprehension of passives. Cognition. 1990 9; 36(3): 227–254. doi.org/10.1016/0010-0277(90)90058-R 2265528 10.1016/0010-0277(90)90058-r

[14] Rowland CF. Explaining errors in children’s questions. Cognition. 2007; 104, 106–134. doi: 10.1016/j.cognition.2006.05.011 16839536 10.1016/j.cognition.2006.05.011

[15] Rowland CF, Noble CH, Chan A. Competition all the way down: how children learn word order cues to sentence meaning In: MacWhinney B, Malchukov A, Moravcsik E, editors. Competing motivations in grammar and usage. Oxford: Oxford University Press; 2014 pp. 127–143 doi: 10.1093/acprof:oso/9780198709848.003.0008

[16] Pine JM. Language-learning environment In: Hogan P, editor. The Cambridge Encyclopedia of the Language Sciences. 2011 pp. 419–420.

[17] Ambridge B, Kidd E, Rowland CF, Theakston A. The ubiquity of frequency effects in first language acquisition. J Child Lang. 2015; 42: 239–273. doi: 10.1017/S030500091400049X 25644408 10.1017/S030500091400049X PMC4531466

[18] Tabor W, Tanenhaus MK. Dynamical models of sentence processing. Cogn Sci. 1999 10; 23(4): 491–515. doi: 10.1016/S0364-0213(99)00013-0

[19] Abbot-Smith K, Behrens H. How known constructions influence the acquisition of other constructions: the German passive and future constructions. Cogn Sci. 2006; 30(6): 995–1026. doi: 10.1207/s15516709cog0000_61 21702844 10.1207/s15516709cog0000_61

[20] Chang F, Dell GS, Bock K. Becoming syntactic. Psychol Rev. 2006; 113(2), 234–272. doi: 10.1037/0033-295X.113.2.234 16637761 10.1037/0033-295X.113.2.234

[21] Fitz H, Chang F. Meaningful questions: The acquisition of auxiliary inversion in a connectionist model of sentence production. Cognition. 2017 6; 166: 225–250. doi: 10.1016/j.cognition.2017.05.008 28582685 10.1016/j.cognition.2017.05.008

[22] Twomey K, Chang F, Ambridge B. Do as I say, not as I do: A lexical distributional account of English locative verb class acquisition. Cogn Psychol. 2014 9; 73: 41–71 doi: 10.1016/j.cogpsych.2014.05.001 24956024 10.1016/j.cogpsych.2014.05.001

[23] Bever TG. The cognitive bias for linguistic structures In: Hayes JR, editor. Cognition and the development of language. New York: Wiley; 1970 pp. 279–362.

[24] Bates E, MacWhinney B. Functionalist approaches to grammar In: Gleitman L, Wanner E, editors. Language acquisition: the state of the art. Cambridge University Press; 1982, pp. 173–218.

[25] Choi Y, Trueswell JC. Children’s (in)ability to recover from garden paths in a verb-final language: evidence for developing control in sentence processing. J EXP CHILD PSYCHOL. 2010; 106(1): 41–61. doi: 10.1016/j.jecp.2010.01.003 20163806 10.1016/j.jecp.2010.01.003 PMC2835831

[26] Hurewitz F, Brown-Schmidt S, Thorpe K, Gleitman L, Trueswell JC. One frog, two frog, red frog, blue frog: Factors affecting children’s syntactic choices in production and comprehension. J Psycholinguist Res. 2000; 29: 597–626. doi: 10.1023/A:1026468209238 11196065 10.1023/a:1026468209238

[27] Trueswell JC, Sekerina I, Hill NM, Logrip ML. The kindergarten-path effect: Studying on-line sentence processing in young children. Cognition; 1999; 73: 89–134 doi: 10.1016/S0010-0277(99)00032-3 10580160 10.1016/s0010-0277(99)00032-3

[28] Kidd E, Stewart A, Serratrice L. Children do not overcome lexical biases where adults do: The role of the referential scene in garden-path recovery. J Child Lang. 2011; 38, 222–234. doi: 10.1017/S0305000909990316 20196901 10.1017/S0305000909990316

[29] de Villiers JG, de Villiers PA. Development of the use of word order in comprehension. J Psycholinguist Res. 1973; 2(4): 331–341. doi: 10.1007/BF01067055 24197918 10.1007/BF01067055

[30] Turner EA, Rommetveit R. The acquisition of sentence voice and reversibility. Child Dev. 1967; 38(3): 649–660 http://www.jstor.org/stable/1127243 6049629

[31] Gertner Y, Fisher C. Predicted errors in children’s early sentence comprehension. Cognition. 2012; 124(1): 85–94. doi: 10.1016/j.cognition.2012.03.010 22525312 10.1016/j.cognition.2012.03.010 PMC3384741

[32] Yuan S, Fisher C, Snedeker J. Counting the nouns: simple structural cues to verb meaning. Child Dev. 2012; 83(4): 1382–1399. doi: 10.1111/j.1467-8624.2012.01783.x 22616898 10.1111/j.1467-8624.2012.01783.x

[33] Mani N, Huettig F. Prediction during language processing is a piece of cake—But only for skilled producers. J Exp Psychol Hum Percept Perform. 2012; 38(4): 843–847. doi.org/10.1037/a0029284 doi: 10.1037/a0029284 22774799 10.1037/a0029284

[34] Huang Y, Zheng X, Meng X, Snedeker J. Children’s assignment of grammatical roles in the online processing of Mandarin passive sentences. J Mem Lang. 2013; 69(4): 589–606. doi: 10.1016/j.jml.2013.08.002 24376303 10.1016/j.jml.2013.08.002 PMC3872120

[35] Dittmar M, Abbot-Smith K, Lieven EVM, Tomasello M. Familiar verbs are not always easier than novel verbs: How German pre-school children comprehend active and passive sentences. Cogn Sci. 2014; 38: 128–151 doi: 10.1111/cogs.12066 23895387 10.1111/cogs.12066

[36] Street J., Dabrowska E. Lexically specific knowledge and individual differences in adult native speakers’ processing of the English passive. Appl Psycholinguist. 2014; 35(1): 97–118. doi.org/10.1017/S0142716412000367

[37] Wechsler D. Wechsler preschool and primary scale of intelligence-WPPSI Psychological Corporation; 1967.

[38] Meints K, Woodford A. Electronic vocabulary database Lincoln UK-CDI Toddlers. 2011. http://www.lincoln.ac.uk/psychology/babylab.htm

[39] Semel E, Wiig EH, Secord W. Clinical Evaluation of Language Fundamentals- Preschool-2. 2nd ed San Antonio, TX: NCS Pearson Inc; 2004.

[40] Dale P, Price T, Bishop D, Plomin R. Outcomes of early language delay: 1. Predicting persistent and transient language difficulties at 3 and 4 years. J Speech Lang Hear Res. 2003; 46: 544–560. doi: 10.1044/1092-4388(2003/044) 14696985 10.1044/1092-4388(2003/044)

[41] Noble CH, Rowland CF, Pine JM. Comprehension of argument structure and semantic roles: Evidence from English-learning children and the forced-choice pointing paradigm. Cogn Sci. 2011; 35(5), 963–982. doi: 10.1111/j.1551-6709.2011.01175.x 21545486 10.1111/j.1551-6709.2011.01175.x

[42] Naigles LR. Children use syntax to learn verb meaning. J Child Lang. 1990; 17: 357–374. doi: 10.1017/S0305000900013817 2380274 10.1017/s0305000900013817

[43] Fernald A, Pinto JP, Swingley D, Weinberg A, McRoberts GW. Rapid gains in speed of verbal processing by infants in the 2nd year. Psychol Sci. 1998; 9(3): 228–231. doi: 10.1111/1467-9280.00044

[44] Arunchalam S, Escovar E, Hansen MA, Waxman SR. Out of sight but not out of mind: 21-month-olds use syntactic information to learn verbs even in the absence of a corresponding event. Lang Cogn Process. 2012; 28: 417–425. doi: 10.1080/01690965.2011.641744 24163490 10.1080/01690965.2011.641744 PMC3805375

[45] Meints K. Typikalizitätseffekte im Erwerb des englischen Passiv. [Typicality effects in the acquisition of the English passive] Opladen: Westdeutscher Verlag; 1999.

[46] Maris E, Oosenveld R. Nonparametric statistical testing of EEG- and MEG-data. J Neurosci Methods. 2007; 164(1): 177–190. doi: 10.1016/j.jneumeth.2007.03.024 17517438 10.1016/j.jneumeth.2007.03.024

[47] von Holzen K, Mani N. Language nonselective lexical access in bilingual toddlers. J EXP CHILD PSYCHOL. 2012; 113: 569–586. doi: 10.1016/j.jecp.2012.08.001 22980955 10.1016/j.jecp.2012.08.001

[48] Dautriche I, Swingley D., Christophe A. Learning novel phonological neighbors: syntactic category matters. Cognition. 2015; 143: 77–86. doi: 10.1016/j.cognition.2015.06.003 26114905 10.1016/j.cognition.2015.06.003 PMC5124220

[49] Chan A, Yang W, Chang F, Kidd E. Four-year-old Cantonese-speaking children’s online processing of relative clauses: A permutation analysis. J Child Lang. 2017 6; doi: 10.1017/S0305000917000198 28606194 10.1017/S0305000917000198

[50] Arunachalam S. Two-year-olds can begin to acquire verb meanings in socially impoverished contexts. Cognition. 2013; 129: 569–573. doi: 10.1016/j.cognition.2013.08.021 24055833 10.1016/j.cognition.2013.08.021

[51] Fernald A, Perfors A, Marchman V. Picking up speed in understanding: speech processing efficiency and vocabulary growth across the second year. Dev Psychol. 2006; 42(1): 98–116. doi: 10.1037/0012-1649.42.1.98 16420121 10.1037/0012-1649.42.1.98 PMC3214591

[52] Grimm H. Sprachentwicklungstest für zweijährige Kinder Diagnose rezeptiver und produktiver Sprachverarbeitungsfähigkeiten. Göttingen: Hogrefe; 2000.

[53] Martin NA, Brownell R. Expressive one-word picture vocabulary test 4: Academic Therapy Publications; 2011.

[54] Dittmar M, Abbot-Smith K, Lieven EVM, Tomasello M. 2;1-year-olds use transitive syntax to make a semantic-role interpretation in a pointing task. J Child Lang. 2011; 38(5): 1109–1123 doi: 10.1017/S0305000910000747 21457589 10.1017/S0305000910000747

[55] Fernandes K, Marcus GF, DiNubila JA, Vouloumanos A. From semantics to syntax and back again: Argument structure in the third year of life. Cognition. 2006; 100(2), B10–B20. doi: 10.1016/j.cognition.2005.08.003 16289066 10.1016/j.cognition.2005.08.003

[56] Baayen RH, Davidson DJ, Bates DM. Mixed-effects modelling with crossed random effects for subjects and items. J Mem Lang. 2008; 59: 390–412. doi: 10.1016/j.jml.2007.12.005

[57] Maratsos M. Children who get worse at understanding the passive: a replication of Bever. J Psycholinguist Res. 1974; 3(1): 65–74.

[58] Allen SE, Crago MB. Early passive acquisition in Inuktitut. J Child Lang. 1996; 23: 129–156. doi: 10.1017/S0305000900010126 8733564 10.1017/s0305000900010126

[59] Messenger K, Branigan H, McLean J. Is children’s acquisition of the passive a staged process? Evidence from six- and nine-year-olds’ production of passives. J Child Lang. 2012; 39(5): 991–1016. doi: 10.1017/S0305000911000377 22182259 10.1017/S0305000911000377

[60] Ambridge B, Bidgood A, Pine JM, Rowland CF, Freudenthal D. Is passive syntax semantically constrained? Evidence from comprehension and grammaticality judgment studies with adults and children. Cogn Sci. 2016; 40(6): 1435–1459 doi: 10.1111/cogs.12277 26607289 10.1111/cogs.12277 PMC4996337

[61] Marchman V, Bates E, Burkardt A, Good A. Functional constraints of the acquisition of the passive: toward a model of the competence to perform. First Lang. 1991; 11(31): 65–92. doi: 10.1177/014272379101103104

[62] Slattery T, Stuart P, Christianson K, Yoshida M, Ferreira F. Lingering misinterpretations of garden path sentences arise from competing syntactic representations. J Mem Lang. 2013; 69(2): 104–120. doi: 10.1016/j.jml.2013.04.001

