# Supplementary material for: Do two and three year old children use an incremental first-NP-as-agent bias to process active transitive and passive sentences?: A permutation analysis
Source: PLoS One. 2017 Oct 19;12(10):e0186129. doi: 10.1371/journal.pone.0186129 (PMC5648151; doi:10.1371/journal.pone.0186129)
Supplement: S1 Appendix — (DOCX) [file pone.0186129.s001.docx]

Appendix A. Visual Stimuli: Video clip pairs and their associated novel verbs used in the test trials of both eye-tracking and pointing tasks

| Verb | Clip 1 | **Clip 2** | |
| --- | --- | --- | --- |
| *‘pogg’* | Agent (A) stands behind P, both side-on to camera. A forces Patient (P) to repeatedly move her /his arms forwards in circles like a pretend steam-train. | P lies on his / her side on floor. A forces P to repeatedly lift his /her upper leg in a stretch towards the ceiling and back down again. | |
| *‘cad’* | P lies stomach-down on floor. A forces P to move each leg repeatedly backwards and forewords in a piston-like fashion. | P kneels and A stands behind P. A forces P to flex her/ his biceps repeatedly. | |
| *‘jit’* | P kneels and A stands behind P. A forces P to repeatedly tilt his / her head on one side and then the other side. | A and P stand facing one another. A repeatedly pulls P’s arms away from him / her and then back towards her chin. | |
| *‘mab’* | A pushes P backwards and forewords repeatedly on a trolley. | A forces P’s chair to turn repeatedly from side to side by using a scarf to swivel the chair. | |
| *‘sem’* | A and P stand facing one another. A repeatedly forces P to flop his / her arms sideways, first to one side and then the other. | | A and P are standing and P is bending down and holding P’s ankle. A forces P to lift one leg repeatedly like a can-can dancer. |
| *‘reft’* | A forces P to repeatedly pat her own head. | | A forces P to rock repeatedly on a rocking-stool by pulling his legs backwards and forewords. |
