# Supplementary material for: Do two and three year old children use an incremental first-NP-as-agent bias to process active transitive and passive sentences?: A permutation analysis
Source: PLoS One. 2017 Oct 19;12(10):e0186129. doi: 10.1371/journal.pone.0186129 (PMC5648151; doi:10.1371/journal.pone.0186129)
Supplement: S2 Appendix — (NB: Order of verb-action pairs was counterbalanced according to Latin squares. Therefore only 1/8 children started with mabbing). (DOCX) [file pone.0186129.s002.docx]

**Appendix B: Sentence stimuli.** (NB: Order of verb-action pairs was counterbalanced according to Latin squares. Therefore only 1/8 children started with mabbing).

| *A: Active version* | *B: Active version* |
| --- | --- |
| The girl is mabbing the boy. Find mabbing! | The boy is mabbing the girl. Find mabbing! |
| The boy is semming the girl. Find semming! | The girl is semming the boy. Find semming! |
| The girl is refting the boy. Find refting! | The boy is refting the girl. Find refting! |
| The boy is pogging the girl. Find pogging! | The girl is pogging the boy. Find pogging! |
| The boy is cadding the girl. Find cadding! | The girl is cadding the boy. Find cadding! |
| The girl is jitting the boy. Find jitting! | The boy is jitting the girl. Find jitting! |
| *A: Passive version* | *B: Passive version* |
| The girl is being mabbed by the boy. Find mabbing! | The boy is being mabbed by the girl. Find mabbing! |
| The boy is being semmed by the girl. Find semming! | The girl is being semmed by the boy. Find semming! |
| The girl is being refted by the boy. Find refting! | The boy is being refted by the girl. Find refting! |
| The boy is being pogged by the girl. Find pogging! | The girl is being pogged by the boy. Find pogging! |
| The boy is being cadded by the girl. Find cadding! | The girl is being cadded by the boy. Find cadding! |
| The girl is being jitted by the boy. Find jitting! | The boy is being jitted by the girl. Find jitting! |
